# The Correlation between the Virus- and Brain Antigen-Specific B Cell Response in the Blood of Patients with Multiple Sclerosis

**DOI:** 10.3390/v8040105

**Published:** 2016-04-23

**Authors:** Marie Wunsch, Christopher Hohmann, Bianca Milles, Christina Rostermund, Paul V. Lehmann, Michael Schroeter, Antonios Bayas, Jochen Ulzheimer, Mathias Mäurer, Süleyman Ergün, Stefanie Kuerten

**Affiliations:** 1 Department of Anatomy and Cell Biology, University of Wuerzburg, Koellikerstr. 6, 97070 Wuerzburg, Germany; chr-rostermund@t-online.de (C.R.); sueleyman.erguen@uni-wuerzburg.de (S.E.); stefanie.kuerten@uni-wuerzburg.de (S.K.); 2 Department of Anatomy I, University of Cologne, Joseph-Stelzmann-Str. 9, 50931 Cologne, Germany; Hohmannc@smail.uni-koeln.de (C.H.); bmilles@smail.uni-koeln.de (B.M.); 3 Cellular Technology Limited, 20521 Chagrin Blvd, Shaker Heights, OH 44122, USA; paul.lehmann@immunospot.com; 4 Department of Neurology, University Hospitals of Cologne, Kerpener Straße 62, 50937 Cologne, Germany; michael.schroeter@uk-koeln.de; 5 Department of Neurology, Klinikum Augsburg, Stenglinstraße 2, 86156 Augsburg, Germany; antonios.bayas@klinikum-augsburg.de; 6 Department of Neurology, Caritas-Krankenhaus Bad Mergentheim, Uhlandstraße 7, 97980 Bad Mergentheim, Germany; jochen.ulzheimer@ckbm.de (J.U.); Mathias.Maeurer@ckbm.de (M.M.)

**Keywords:** B cells, CMV, EBV, ELISPOT, MS

## Abstract

There is a largely divergent body of literature regarding the relationship between Epstein-Barr virus (EBV) infection and brain inflammation in multiple sclerosis (MS). Here, we tested MS patients during relapse (*n* = 11) and in remission (*n* = 19) in addition to *n* = 22 healthy controls to study the correlation between the EBV- and brain-specific B cell response in the blood by enzyme-linked immunospot (ELISPOT) and enzyme-linked immunosorbent assay (ELISA). Cytomegalovirus (CMV) was used as a control antigen tested in *n* = 16 MS patients during relapse and in *n* = 35 patients in remission. Over the course of the study, *n* = 16 patients were untreated, while *n* = 33 patients received immunomodulatory therapy. The data show that there was a moderate correlation between the frequencies of EBV- and brain-reactive B cells in MS patients in remission. In addition we could detect a correlation between the B cell response to EBV and disease activity. There was no evidence of an EBV reactivation. Interestingly, there was also a correlation between the frequencies of CMV- and brain-specific B cells in MS patients experiencing an acute relapse and an elevated B cell response to CMV was associated with higher disease activity. The trend remained when excluding seronegative subjects but was non-significant. These data underline that viral infections might impact the immunopathology of MS, but the exact link between the two entities remains subject of controversy.

## 1. Introduction

Multiple sclerosis (MS) is an autoimmune disease caused by the infiltration of autoreactive lymphocytes into the central nervous system (CNS). How these lymphocytes become activated against the CNS remains unclear. Several infectious agents have been postulated to trigger the development of autoimmune diseases. The role of Epstein-Barr virus (EBV) in the pathogenesis of MS has been investigated in several clinical trials [1,2,3,4]. EBV, a B-lymphotropic herpes virus, is present in all populations and infects over 90% of individuals before adulthood [5,6].

Once infected, the virus is able to persist in the host in a dormant state for an entire lifetime. Periodically, the virus can be reactivated, at which time the viral replication cycle is initiated and lymphocytes are exposed to viral antigens [7]. Activation and expansion of EBV-specific lymphocytes will then force the virus back into its latent state. If primary infection is delayed beyond the first decade of life, it often results in infectious mononucleosis (IM), a self-limiting disease with flu-like symptoms. Thacker *et al.* postulated that EBV infection, which manifests itself as IM in adolescents and young adults, constitutes a risk factor for MS [8]. Furthermore, one study showed evidence of EBV infection in a substantial proportion of B cells and plasma cells found in *post mortem* MS brain tissue [3]. Moreover, there seems to be an increased risk of developing MS when high titers of anti-EBV antibodies are present in the serum [9].

Thus far, the analyses of a correlation between brain reactivity and a positive EBV response were limited due to the fact that there were no reliable parameters reflecting cellular autoimmunity to CNS antigens in MS. In several trials the EBV serum antibody titer has been correlated with clinical and magnetic resonance imaging (MRI) evidence of disease activity [10,11]. The major drawback of these studies was that neither MRI lesions nor the Expanded Disability Status Scale (EDSS) were reflective of the cellular immunity to brain antigens. We have recently introduced an enzyme-linked immunospot (ELISPOT) assay for the detection of brain-specific B cells in the blood of patients with MS. These B cells only occurred in patients with clinically isolated syndrome or definite MS and were absent in healthy donors and in patients with other inflammatory and non-inflammatory neurological diseases as well as other autoimmune disorders [12,13]. In addition, the presence of directly *ex vivo* detectable brain antigen-specific B cells during relapse was associated with a significantly increased risk of the development of a subsequent relapse within the next few months [13].

In the following, we used this assay to study the correlation between the EBV-, Cytomegalovirus (CMV)- and brain-specific B cell response as detected in the blood of patients with MS. The data show that there was no difference in the EBV-specific B cell response in the blood or the previous viral reactivation status comparing healthy donors and MS patients. Along these lines, the B cell response status to EBV did not have a direct clinical impact on the course and severity of established MS. Interestingly, however, there was an association between the frequencies of CMV- and brain-reactive B cells in the blood and disease activity in MS.

## 2. Materials and Methods

### 2.1. Subjects

Forty-one patients that were diagnosed with MS according to the 2005 or 2010 McDonald criteria [14,15], respectively, were included in the study. Sixteen of these patients were undergoing an acute MS relapse. Aggravation of persistent disabilities or new clinical symptoms were present for at least 24 h. Exclusion criteria comprised severe accompanying systemic or psychiatric disorders as well as a history of other autoimmune diseases. Subjects who had undergone plasmapheresis or received anti-B cell therapy were also excluded. Details on all patients and healthy control subjects are provided in Table 1 and Table 2. In addition, Table 3 provides information on the immune modulatory treatment of the MS patients included in the study. The research protocol was approved by the institutional ethics committee of the University of Cologne and the Bayerische Landesärztekammer (approval numbers 10–221 and mb BO 14043). For the evaluation of disease severity the EDSS was used [16]. All patients gave written informed consent and were recruited from a MS clinical care unit at the Department of Neurology of the University Hospitals of Cologne, the Department of Neurology, Klinikum Augsburg, Germany and the Department of Neurology, Caritas-Krankenhaus Bad Mergentheim, Germany. Peripheral blood mononuclear cells (PBMC) and plasma samples from healthy controls were obtained from *n* = 22 volunteers at the participating institutions after written informed consent.

### 2.2. ELISPOT

**Figure 1:**
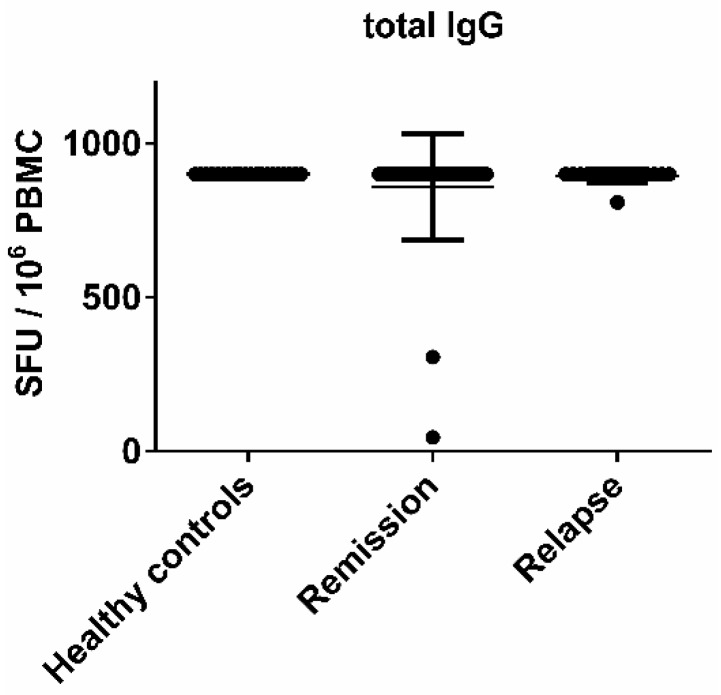
No significant difference in the numbers of immunoglobulin G (IgG)-secreting B cells between healthy controls and multiple sclerosis (MS) patients. Peripheral blood mononuclear cells (PBMC) were obtained from healthy controls, MS patients in remission and during an acute relapse. The numbers of total IgG secreting B cells in the polyclonally stimulated PBMC population are shown. Bars represent median values and standard deviations.

PBMC were isolated from the blood by Ficoll-Paque (GE Healthcare Europe GmbH, Freiburg, Germany) density gradient centrifugation. For polyclonal stimulation of B cells, PBMC were cultured for 96 h prior to the ELISPOT assay at a concentration of 3 × 10^6^ cells/mL in complete Roswell Park Memorial Institute (RPMI)-1640 medium (Lonza, Cologne, Germany) that was supplemented with R-848 at 2.5 μg/mL (Enzo Life Sciences, Inc., Farmingdale, NY, USA), interleukin (IL)-2 at 15 ng/mL (Peprotech, Hamburg, Germany) and 1 μmol β-mercaptoethanol (Sigma, Schnelldorf, Germany). Polyclonal stimulation with R-848 and IL-2 has previously been shown to be a simple method for the selective activation of memory B cells [15]. Complete RPMI medium consisted of RPMI-1640 containing 10% fetal bovine serum (FBS) (Biochrom, Berlin, Germany), 1% L-glutamine (Sigma) and 1% penicillin/streptomycin (Sigma). The plates were coated overnight with whole normal human brain lysate, which was isolated from fresh frozen tissue (30 μg/mL; Novus Biologicals, Littleton, CO, USA), human CMV grade 2 antigen (100 µg/mL; Microbix Biosystems Inc., Mississauga, ON, Canada) or prolyl 3-hydroxylase 3 (P3H3) cell extract EBV antigen (50 µg/mL; Microbix Biosystems Inc., Mississauga, ON, Canada). Coating with anti-human Igκ at 10 μg/mL (SouthernBiotech, Birmingham, AL, USA) served as a positive control. Plates were blocked with 10% FBS in sterile phosphate-buffered saline (PBS) at room temperature for 2 h. Each sample was plated in duplicates with one million polyclonally stimulated PBMC per well for human brain lysate and CMV and 500,000 polyclonally stimulated PBMC per well for EBV, which we found to be the optimal cell concentration in titration experiments. Biotinylated anti-human immunoglobulin (Ig)G (Hybridoma Reagent Laboratory, Baltimore, MD, USA) diluted in 1% bovine serum albumin (BSA) solution was used as a detection antibody at 0.2 μg/mL. Figure 1 shows that the total IgG production as measured by ELISPOT was comparable between all individuals included in the study. Therefore, we assume that the number of plated antibody secreting cells was similar in each donor. All plates were developed with Vector Blue substrate (Vector Laboratories, Burlingame, CA, USA). Spots were counted on an ImmunoSpot^®^ Series 6 Analyzer (Cellular Technology Limited, Shaker Heights, OH, USA).

### 2.3. ELISA

ELISA plates (Thermo Scientific, Schwerte, Germany) were coated overnight with whole normal human brain lysate (10 μg/mL; Novus Biologicals), human CMV grade 2 antigen (10 µg/mL; Microbix Biosystems Inc.), P3H3 cell extract EBV antigen (10 µg/mL; Microbix Biosystems Inc.), active EBV (EA regions) (10 µg/mL; Abcam; Cambridge, UK), EBV nuclear antigen full length protein EBNA1 (10 µg/mL; Abcam), or anti-human Igκ (2.5 μg/mL; SouthernBiotech), respectively, all diluted in PBS or with PBS alone. As for the ELISPOT assay, all antigens were titrated to their optimal concentration for use in the antibody ELISA. Plates were blocked with 10% FBS in PBS containing 0.05% Tween 20 at room temperature for 2 h. The plates were incubated with plasma at 4 °C overnight. All plasma samples were diluted 1:400 in 10% FBS solution containing 0.05% Tween 20 detergent. Biotinylated anti-human IgG (Hybridoma Reagent Laboratory) diluted in 0.5% FBS/0.05% Tween 20 solution was used as a detection antibody at 0.05 μg/mL. All plates were developed with tetramethylbenzidine substrate (eBioscience, Frankurt, Germany) after incubation with streptavidin-horseradish peroxidase (eBioscience) at 1:1000 dilution. The reaction was stopped with 0.16 M sulphuric acid and the optical density (OD) in the wells was read at 450 nm using a Perkin Elmer Victor 3 1420 Multilabel Counter and Wallac 1420 software version 3.00 revision 5.

### 2.4. Statistical Analysis

ELISPOT results were compared between the test subject groups with the use of the Wilcoxon rank-sum or Kruskal-Wallis test followed by a Gabriel’s *post hoc* test. For comparing the mean spot size differences between brain- and virus-specific B cell spots the Wilcoxon rank-sum test was used. Fisher’s exact test was used to assess statistical differences in prevalence rates. *p*-values that were equal to or less than 0.05 were considered to indicate statistical significance. The correlation between the frequencies of brain- and virus-specific B cells was calculated using Spearman’s rank correlation. Statistical analyses were performed using Prism 6 (GraphPad Software, Inc., La Jolla, CA, USA) and SPSS Statistics 22 (IBM, New York, NY, USA).

## 3. Results

PBMC were isolated from healthy controls and MS patients experiencing an acute relapse or in remission, which was, on average, four months after a relapse. PBMC were polyclonally stimulated with R-848, IL-2 and β-mercaptoethanol for 96 h to activate resting B cells [17]. The numbers of EBV- and brain-specific B cells were determined using ELISPOT and plasma antibody titers were quantified in ELISA assays. As a control, we also investigated the cellular and plasma antibody response to CMV.

### 3.1. There Was No Significant Difference in the EBV-Specific B Cell Activity Comparing Healthy Controls and MS Patients

**Figure 2:**
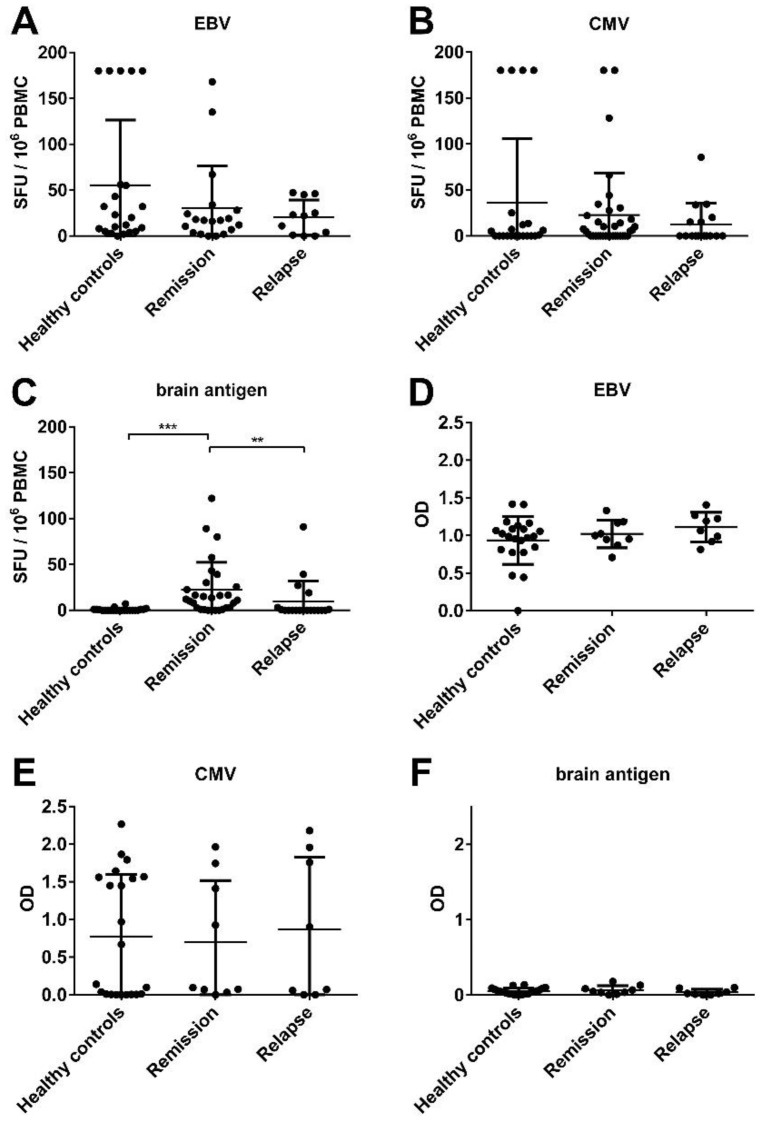
No significant difference in the virus-, but in the brain-specific B cell activity between healthy controls and MS patients. PBMC and plasma were obtained from healthy controls, MS patients experiencing an acute relapse and MS patients in remission. The numbers of Epstein-Barr virus (EBV)- (**A**); Cytomegalovirus (CMV)- (**B**) and brain antigen-specific (**C**) B cells in the polyclonally stimulated PBMC population are shown in addition to the plasma antibody level for EBV (**D**); CMV (**E**) and brain antigen (**F**) for each individual donor in the specified groups. Bars represent median values and standard deviations.

The numbers of EBV- and brain-specific B cells were quantified in *n* = 11 MS patients experiencing an acute relapse and *n* = 19 MS patients in remission. In addition, CMV- and brain-specific B cell numbers were quantified in *n* = 16 MS patients during relapse and *n* = 35 MS patients in remission. We also measured the plasma antibody titers in *n* = 10 patients. The data were compared to a total of *n* = 22 healthy controls. An EBV response was detectable in the ELISPOT in 95.5% of the healthy controls, 81.8% of MS patients experiencing an acute relapse and in 89.5% of MS patients during remission. CMV-specific B cells were detectable in 50% of the healthy controls, 37.5% of MS patients experiencing a relapse and in 54.3% of MS patients in remission. In all groups the frequencies of virus-specific B cells showed a wide distribution ranging from 0 to 180 in 10^6^ stimulated PBMC. Overall, there was no significant difference in the EBV- or CMV-specific B cell frequencies in healthy controls and MS patients (Figure 2A,B). As expected from our previous studies [12,13], brain-specific B cells were barely detectable in healthy controls (Figure 2C). In contrast, MS patients showed numbers between 0 and 122 brain-specific B cells. 65.5% of the MS patients in remission and 21% of the patients experiencing an acute relapse were B cell responders to brain proteins in the ELISPOT (Figure 2C). In *n* = 2 patients no B cell response to brain proteins could be detected during the relapse, but over time there was a conversion to a B cell positive response in remission. In accordance with the ELISPOT data, plasma antibody titers to EBV and CMV were comparable in both healthy controls and MS patients (Figure 2D,E). As we have shown before [13], in the ELISA assay there was rarely any plasma antibody response to brain antigens in MS patients (Figure 2F).

### 3.2. The Morphology of Spots Produced by Virus-Specific B Cells Was Similar in Healthy Controls and MS Patients, Hence There Was No Difference in Productivity and the Kinetics of Cell Secretory Activity

**Figure 3:**
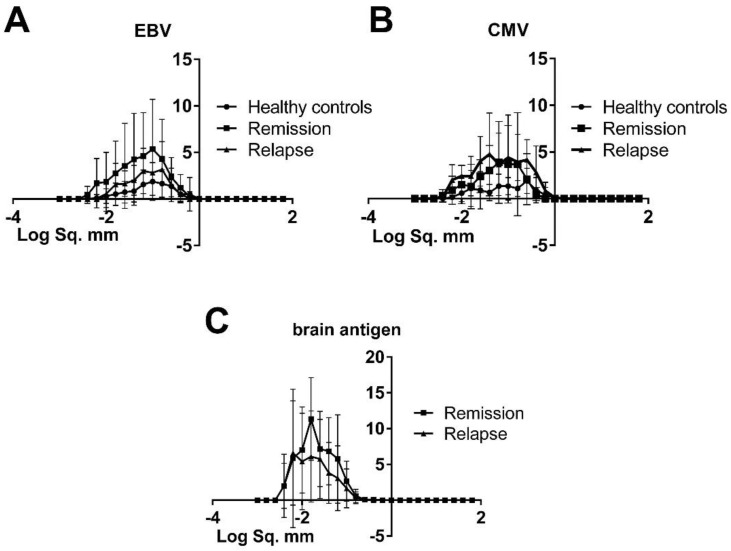
Similar morphology of spots produced by virus-specific B cells in healthy controls and MS patients. The line plots represent the mean spot sizes and standard deviations for EBV (**A**); CMV (**B**) and brain antigen (**C**) detected in the ELISPOT in healthy controls and MS patients experiencing a relapse and in remission. Spot sizes for brain antigen are displayed for MS patients only since spots were absent in healthy controls.

The analysis of spot sizes in the ELISPOT can provide important information on the quantity of antibodies produced by individual cells as well as the kinetics of the secretory process [18]. To this end, we compared the morphology of spots produced by virus-specific B cells in healthy controls and MS patients using a specialized ImmunoSpot™ image analysis software. Furthermore, we compared the spots produced by brain-specific B cells of MS patients experiencing a relapse with those produced in remission. As shown in Figure 3, there were no significant differences in size or morphology of spots produced by virus-specific B cells in healthy controls and MS patients. Typically virus-specific spots were large in size and well-defined. The mean spot size for EBV was 0.155 ± 0.077 mm^2^ in healthy controls, 0.157 ± 0.107 mm^2^ in MS patients during relapse and 0.149 ± 0.074 mm^2^ during remission. For CMV we detected a mean spot size of 0.119 ± 0.107 mm^2^ in healthy controls, 0.159 ± 0.047 mm^2^ in MS patients experiencing an acute relapse and 0.154 ± 0.097 mm^2^ in MS patients in remission. The morphology of spots produced by brain-reactive B cells in MS patients experiencing a relapse compared to patients in remission was also similar (compare 0.022 ± 0.014 mm^2^ to 0.034 ± 0.021 mm^2^). However, brain-specific B cell spots were significantly smaller than virus-specific spots (compare 0.029 ± 0.02 to 0.147 ± 0.091; *p* < 0.001).

### 3.3. There Was a Correlation between the Frequencies of Virus- and Brain-Reactive B Cells in the Blood of MS Patients

**Figure 4:**
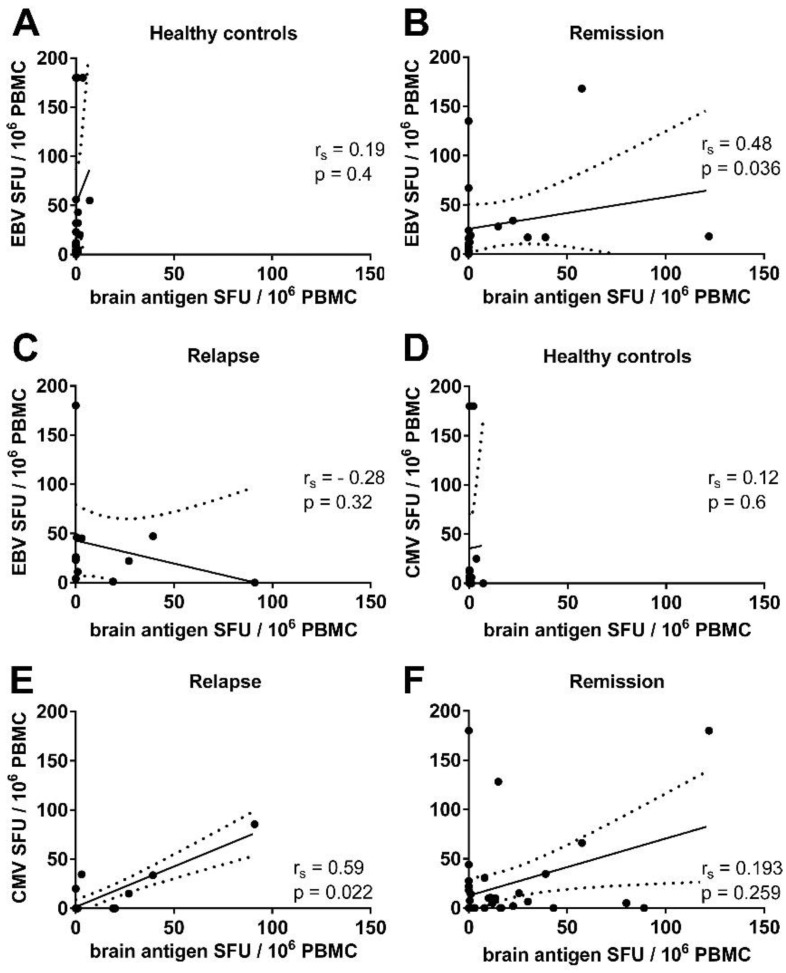
Correlation between the frequencies of virus- and brain antigen-specific B cells in the blood of MS patients. Spearman’s rank correlation (r*_s_*) representing the ratio between EBV and brain antigen-specific B cell numbers in the polyclonally stimulated PBMC population of healthy controls (**A**); MS patients in remission (**B**) and MS patients experiencing an acute relapse (**C**); (**D**–**F**) Correlation between CMV and brain antigen-specific B cell numbers in healthy controls (**D**); MS patients in remission (**E**) and MS patients experiencing an acute relapse (**F**). Lines represent the linear regression, and dotted lines the 95% confidence interval.

To investigate the relationship between virus and brain reactivity we correlated B cell reactivity to EBV with the response to brain antigens in *n* = 19 MS patients in remission and *n* = 11 MS patients experiencing an acute relapse. In addition, we correlated the B cell response to CMV with the numbers of brain antigen-specific B cells in *n* = 35 patients in remission and *n* = 16 MS patients experiencing an acute relapse. Due to the fact that healthy controls barely showed any B cell response to brain antigens, there was no correlation between virus and brain reactivity (Figure 4A,D). However, we could detect a significant correlation between the frequencies of EBV- and brain-reactive B cells in MS patients in remission, but not in MS patients experiencing an acute relapse (Figure 4B,C). In three MS patients no brain-reactive B cells could be detected, but EBV-specific B cells ranged from 19 to 135 cells in 10^6^ stimulated PBMC from these patients. There was also a significant correlation between the frequencies of CMV- and brain-specific B cells in MS patients experiencing an acute relapse (Figure 4E). There was no significant correlation between the numbers of CMV- and brain-specific B cells in MS patients in remission, e.g., a high frequency of brain-reactive B cells was not linked to a high frequency of CMV-specific B cells (Figure 4F).

### 3.4. An Acute MS Relapse Was Not Associated with any Change in the Virus-Specific B Cell Activity, but with a Decrease in Brain-Reactive B Cell Responses Compared to the Remission

**Figure 5:**
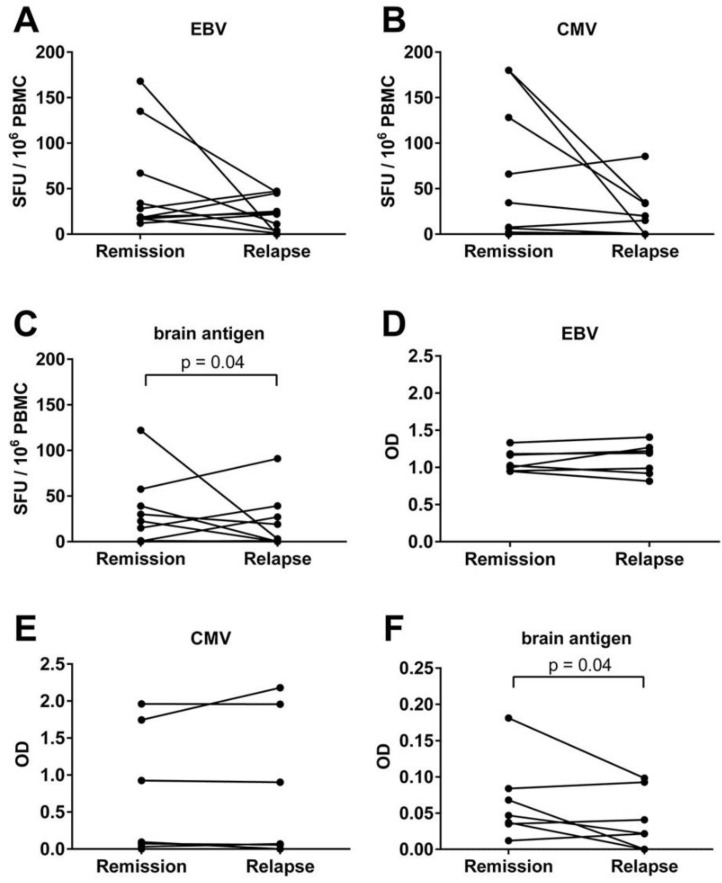
Similar magnitude of the virus-specific B cell response in relapse and remission. The frequencies of EBV- (**A**) CMV- (**B**) and brain antigen-specific (**C**) B cells and the plasma antibody levels for EBV (**D**), CMV (**E**) and brain antigen (**F**) were detected in *n* = 10 MS patients experiencing a relapse and in the same MS patients in remission. The results for the individual donors are displayed.

We analyzed if an acute MS relapse was associated with a shift of the virus- or brain-specific B cell activity. To this end, we quantified the virus- and brain antigen-specific B cell response in *n* = 10 MS patients experiencing an acute relapse and the same 10 patients in remission. In most patients the virus-specific B cell response was stable (Figure 5). Nevertheless, an up to five-fold increase and an up to 168-fold decrease of EBV-specific B cell numbers in remission compared to the relapse could be detected in individual patients (Figure 5A). Similar diversities were observed when comparing the CMV-specific B cell frequencies in remission and relapse (Figure 5B). Two patients displayed a high CMV response with 180 CMV-specific B cells in remission and a drop to 34.5 or 0 CMV-specific B cells, respectively, during relapse. On the contrary, other patients showed an up to two-fold decrease of the CMV-specific response in remission compared to the relapse (Figure 5B). The variations in virus-specific B cell numbers in individual patients detected by ELISPOT were not evident when measuring CMV- and EBV-specific plasma antibody levels. The optical density (OD) was remarkably constant in remission and relapse (Figure 5D,E). Overall, we could not detect any significant difference in the CMV- or EBV-specific B cell frequencies and antibody titers in MS patients experiencing an acute relapse compared to the remission. In contrast, a significant difference in the brain-reactive B cell frequencies in MS patients experiencing a relapse compared to the remission was observed (*p* = 0.04). On average there was a decrease from 27.05 spot forming units (SFU) in remission to 20 SFU during relapse (Figure 5C).

### 3.5. There Was a Trend for a Correlation between the Frequencies of CMV- and Brain-Reactive B Cells in the Blood of MS Patients Experiencing an Acute Relapse when Excluding CMV Seronegative Subjects

**Figure 6:**
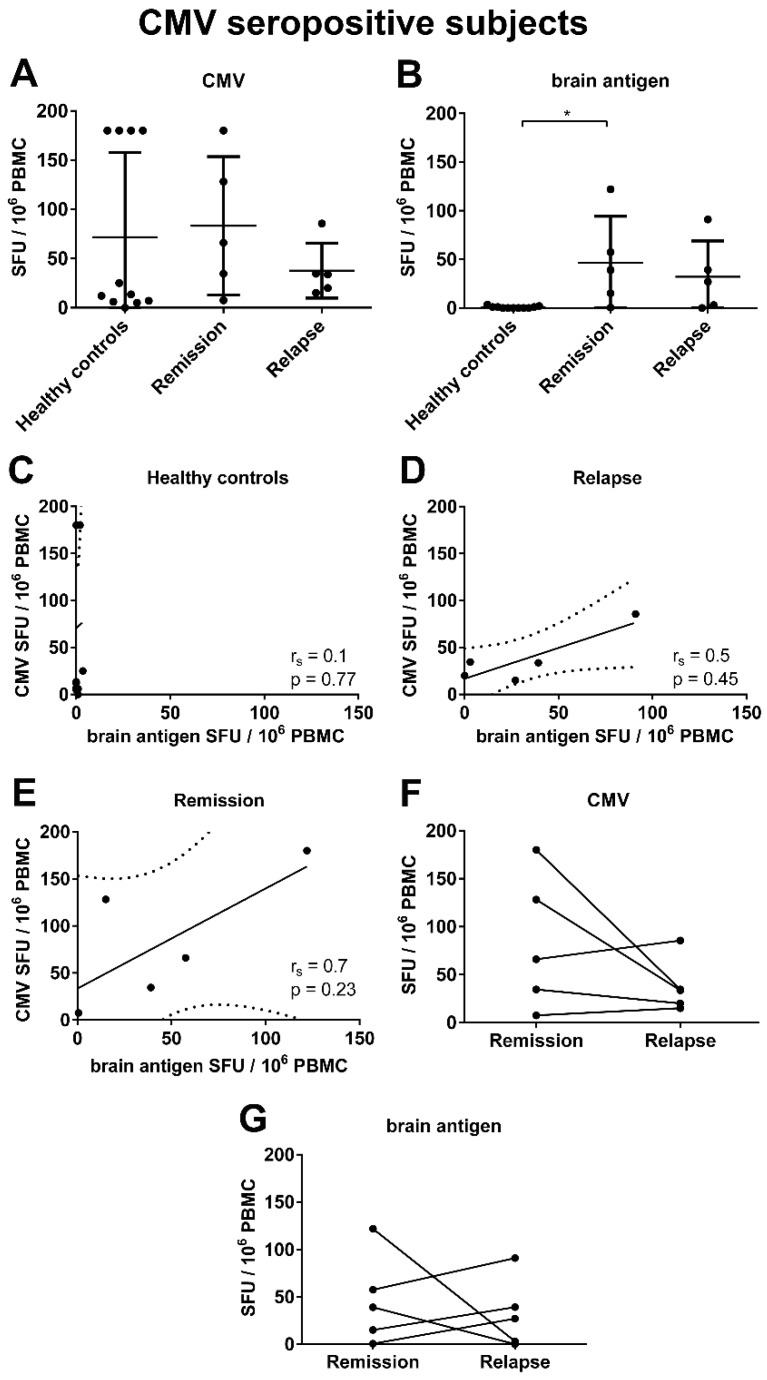
CMV-specific and brain antigen-specific B cell frequencies and correlation analysis with CMV seropositive subjects only. The frequencies of CMV- (**A**) and brain antigen-specific (**B**) B cells were determined in *n* = 11 healthy controls and *n* = 5 MS patients experiencing a relapse and in the same MS patients in remission for CMV seropositive subjects only. Spearman’s rank correlation (r*_s_*) analysis for CMV and brain antigen-specific B cell numbers in healthy controls (**C**), MS patients experiencing an acute relapse (**D**) and MS patients in remission (**E**); lines represent the linear regression, and dotted lines the 95% confidence interval. There was a similar magnitude of the CMV- (**F**) and brain antigen-specific (**G**) B cell response in relapse and remission. The results for the individual donors are displayed.

It could be argued that the viral seropositivity status might have had an effect on our results presented so far. While all subjects were seropositive for EBV, 11 of 22 healthy controls and 5 of 11 MS patients were positive for CMV. There was no significant difference in the CMV-specific B cell frequencies in CMV seropositive healthy controls and MS patients. Brain-specific B cells were barely detectable in healthy controls, but could be detected in MS patients (Figure 6B,C). Due to the low number of CMV seropositive MS patients experiencing an acute relapse, we could not detect a significant correlation between the frequencies of CMV- and brain antigen-specific B cells in the blood (Figure 6D). However, the tendency remained similar to the data obtained when including all subjects with a Spearman’s rank correlation of 0.5. Furthermore, there was a similar magnitude of the CMV-specific and brain antigen-specific B cell response in relapse and remission after excluding seronegative subjects (Figure 6F,G).

### 3.6. MS Is Not Associated with the Reactivation of a Latent EBV Infection

**Figure 7:**
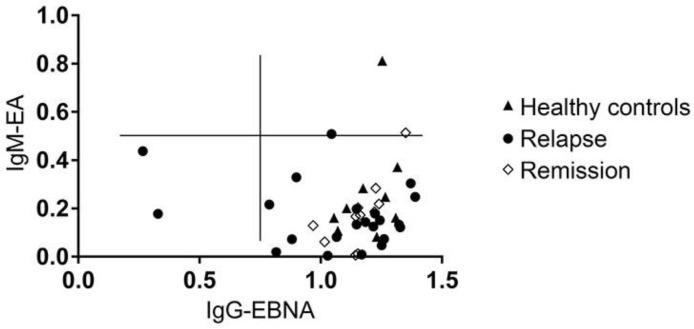
No association between MS and the reactivation of a latent EBV infection. Scatter plot for EBV nuclear antigen (EBNA) IgG and EBV early antigen (EA) IgM plasma titers in the different subgroups. Results for individual donors are displayed as circles.

Reactivation of latent EBV infection was defined as the simultaneous seropositivity to IgM directed against EBV early antigen (IgM-EA) and IgG directed against EBV nuclear antigen (IgG-EBNA) [19]. A high IgM-EA titer associated with a high IgG-EBNA titer was evident in one healthy control (IgM-EA OD: 0.80; IgG-EBNA OD: 1.25), in one MS patient experiencing an acute relapse (IgM-EA OD: 0.51; IgG-EBNA OD: 1.0) and in one MS patient in remission (IgM-EA OD: 0.50; IgG-EBNA OD: 1.35). The plasma IgM-EA titers in the other MS patients and healthy controls were below an OD of 0.49. Hence, there was no evidence of EBV reactivation (Figure 7).

### 3.7. There Was a Correlation between the Virus-Specific B Cell Response and Clinical Disease Parameters

To test whether an elevated B cell response to CMV or EBV was associated with clinical disease parameters, we correlated the virus-specific B cell frequencies with disease activity, age at time of diagnosis and the EDSS. We could detect a significant correlation between EBV-specific spot numbers and disease activity in MS patients in remission (Table 4). Furthermore, there was a correlation between the B cell response to CMV and disease activity in MS patients in relapse and remission.

## 4. Discussion

The foundations of an efficient and functional immune system are both high diversity and specificity of the lymphocyte pool. A side effect of the high diversity is that besides pathogen-specific cells, also autoreactive lymphocytes are released from the bone marrow and thymus. Circulating autoreactive B cells were found in healthy individuals without any history of an autoimmune disease [20]. The question, which has remained unclear, is which mechanisms trigger the activation of these naïve autoreactive lymphocytes and initiate the development of an autoimmune disease only in some individuals compared to those individuals whose autoreactive cells remain in a dormant state and will never cause tissue damage. Various studies have focused on EBV and CMV as potential triggers of MS [1,2,3,4,21,22,23]. There are several possible mechanisms including molecular mimicry, epitope spreading or bystander activation explaining the link between an anti-viral immune response and MS [24]. These mechanisms linking viral infections to MS pathology are largely hypothetical [25]. Molecular mimicry occurs when peptides from pathogens share structural similarities with self-antigens of the CNS, which leads to the activation of autoreactive lymphocytes. Infection with various pathogens, each with its individual molecular resemblance to a CNS antigen, may explain the inability of investigators to link one specific virus to MS [26]. Wucherpfennig and Strominger showed that EBV peptides could activate myelin basic protein (MBP)-specific T cell clones isolated from the blood of MS patients [27]. Bystander activation is based on the fact that viral infections lead to inflammation and activation of antigen-presenting cells (APC) such as dendritic cells. These activated APC could potentially activate autoreactive lymphocytes, which may then initiate autoimmune diseases [28]. However, at the same time, the hypothesis of epitope spreading and bystander activation highlights the notion that the immunopathology may diverge in disease evolution and manifested MS.

The consensus of these hypotheses is that there is a correlation and/or cross-reactivity between brain- and virus-specific lymphocytes. In this study we used the ELISPOT approach to detect brain- and virus-specific B cells in the blood of MS patients and healthy controls. To our knowledge this is the first study comparing the B cell response to EBV, CMV and brain antigens using a cell-based assay. In this assay we used an EBV lysate as antigen mix to coat the plates. There are studies pointing out that EBV lysates are more immunogenic than EBV-encoded nuclear antigen 1 (EBNA-1). Loebel *et al.* could show that EBV lysate induced production of several cytokines in particular interferon (IFN)-γ in whole blood in 50% of chronic fatigue syndrome (CFS) patients. Using EBNA-1 protein for stimulation, no patient showed a detectable IFN-γ response [29]. We suggest that the use of EBV lysate has the advantage that it covers various immunogenic EBV proteins, which minimizes the risk of false negative results.

Our data demonstrate a correlation between EBV-specific and brain-reactive B cells in the blood of MS patients in remission. We also could detect a significant association between the frequencies of EBV-specific B cells and the disease activity in MS patients.

These results go in line with earlier studies. Latham *et al.* postulated a correlation between the frequencies of EBV-specific T cells in the blood and the number of active lesions on MRI scans [30] and Levin *et al.* found high serum levels of IgG antibodies to EBV to be a strong predictor of MS [31]. Serafini *et al.* observed the presence of EBV-infected B cells in the brain in almost 100% of MS cases in addition to EBV reactivation in plasma cells in acute MS lesions and ectopic B cell follicles [3]. These findings support a role for EBV infection in B cell activation in the MS brain, which may contribute to the disruption of B cell tolerance [32]. Still, EBV-triggered B cell activation may be rather a consequence and not the cause of B cell activation paralleling polyclonal activation of serum antibodies against several viral agents in MS that is referred to as MRZ (measles-rubella-zoster) reaction. The increased viral load in the brain as compared to the blood suggested that a locally dysregulated viral infection could support the autoimmune response and tissue damage in the brain [3]. These earlier results may explain why we could not detect increased numbers of EBV-specific B cells and antibody titers in MS patients experiencing an acute relapse due to the fact that EBV-specific cells might accumulate in the brain during acute inflammation. Under these conditions EBV-specific B cells circulating as memory B cells could become readily detectable in the blood during remission, which was the case in our study.

The findings by Serafini *et al.* are in strong contrast to later studies that rarely detected EBV-infected B cells in MS brains. Willis *et al.* studied a large cohort of MS specimens containing white matter lesions with parenchymal and meningeal B cell infiltrates and they could not detect EBV in any of the specimens using multiple techniques including *in situ* hybridization, immunohistochemistry and two independent real-time PCR approaches [33]. Moreover, Sargsyan *et al.* failed to identify EBV infection in cerebrospinal fluid (CSF) B and plasma blast cell populations and EBV-specific transcripts were not detected in MS lesions. In addition, the extent of intrathecal anti-EBV antibody synthesis in patients with MS did not differ from that in non-MS inflammatory CNS disease patients [34]. Overall, there is a largely divergent body of literature regarding the relationship between EBV and MS brain inflammation.

The influence of CMV on MS is also disputed. There are studies supporting a detrimental role of CMV as a trigger of MS, whereas most of the studies describe CMV infection as disease limiting. CMV has been found in demyelinating lesions and a T cell response against CMV epitopes has been observed within CD8+ cells derived from chronic inflammatory lesions [35]. Other researchers found that the time to relapse decreased and the number of relapses increased with anti-CMV IgG positivity [36]. In this study we were able to show that there was a significant correlation between the CMV- and brain antigen-specific B cell response in MS patients experiencing an acute relapse. Furthermore, an elevated B cell response to CMV correlated with a higher disease activity. In earlier studies we demonstrated that treatment-related effects had no impact on the ELISPOT results since the number of brain antigen-specific B cell positive MS patients was independent of the treatment status [12]. However, to further analyze the impact of the immune modulatory treatment on our results, we correlated the frequencies of CMV-specific B cells with disease activity in patients in remission who were untreated (*n* = 11). The Spearman’s rank correlation was 0.69 and the *p*-value 0.035. This shows that the treatment does not significantly impact the results of the correlation analysis.

Earlier studies detected a correlation between CMV serum antibody titers and an increased MS disease risk. Sundqvist *et al.* could show that CMV seropositivity was associated with a decreased risk of developing MS [37]. Additionally, Zivadinov *et al.* investigated an association between clinical and MRI measures of disease activity and the presence and titer of IgG antibodies against CMV in *n* = 140 patients with definite MS and *n* = 131 healthy controls [38]. In their study there was an association between antibody positivity against CMV, a higher titer and better clinical and MRI outcomes [38]. Limitations and a reason for the discrepancy in the results of the earlier studies could be the low sensitivity, which is due to the measurement of serum antibody titers rather than detecting virus-specific cells, and the lack of the technique reflecting cellular autoimmunity to brain antigens. Along these lines, in an independent currently ongoing study we were able to show that measurements of serum antibodies frequently failed to reveal CMV exposure in humans, which may be better identified by direct detection of CMV-specific memory lymphocytes [39]. In the current study the tendency for a correlation between the CMV- and brain antigen-specific B cell response in MS patients experiencing a relapse remained similar when excluding CMV seronegative subjects. However, the correlation was not significant most likely due to the low number of CMV seropositive MS patients.

## 5. Conclusions

The role of EBV infection in MS is a widely debated topic of high clinical relevance. Our study provides small additional support to the theory of a link between EBV infection and MS. However, the two main limitations of our study are that the sample size was limited and CSF was not available to us. Interestingly, however, we noted a correlation between the frequencies of CMV- and brain antigen-specific B cells in the blood of MS patients. These data underline that viral infections might still play an important role in the immunopathology of MS, but the exact link between the two entities remains subject of controversy.

## Figures and Tables

**Table 1:** Summary of healthy control demographics.

No. of healthy controls	22
Female sex—No. (%)	13 (59.1)
Mean (SE) age—years	34.1 (12.2)

**Table 2:** Summary of multiple sclerosis (MS) patient demographics.

No. of patients	41
Female sex—No. (%)	25 (61)
Mean (SE) age—years	38 (10.8)

**Table 3:** Immune modulatory treatment of MS patients included in the study.

Treatment	*n*^a^	CMV Positive	EBV Positive	Brain Antigen Positive
untreated	16	9/16 (56.3%)	8/11 (72.7%)	5/16 (31.3%)
IFN-β	14	4/14 (28.6%)	5/5 (100%)	8/14 (57.1%)
glatiramer acetate	5	4/5 (80%)	1/1 (100%)	3/5 (60%)
fumaric acid	1	0/1 (0%)	n. a.	0/1 (0%)
natalizumab	8	4/8 (50%)	7/7 (100%)	1/8 (12.5%)
fingolimod	1	1/1 (100%)	1/1 (100%)	0/1 (0%)
mitoxantrone	2	0/2 (0%)	2/2 (100%)	1/2 (50%)
rituximab	2	0/2 (0%)	0 (0%)	0/2 (0%)

^a^ The n-number is higher than the total number of patients included because some of the patients switched therapy during the course of the study.

**Table 4:** Correlation between virus-specific SFU/10^6^ PBMC with clinical parameters.

Correlation	Relapse	Remission
EBV—Disease activity ^a^	r*_s_* = 0.39; *p* = 0.24 *(n = 11)*	r*_s_* = 0.58; *p* = 0.009 *(n = 19)*
EBV—Age at time of diagnosis	r*_s_* = −0.31; *p* = 0.32 *(n = 11)*	r*_s_* = 0.023; *p* = 0.93 *(n = 19)*
EBV—Remission EDSS	r*_s_* = 0.053; *p* = 0.88 *(n = 11)*	r*_s_* = −0.05; *p* = 0.84 *(n = 19)*
CMV—Disease activity ^a^	r*_s_* = 0.68; *p* = 0.004 *(n = 16)*	r*_s_* = 0.39; *p* = 0.02 *(n = 35)*
CMV—Age at time of diagnosis	r*_s_* = −0.2; *p* = 0.12 *(n = 16)*	r*_s_* = 0.01; *p* = 0.94 *(n = 35)*
CMV—Remission EDSS	r*_s_* = −0.18; *p* = 0.12 *(n = 16)*	r*_s_* = 0.15; *p* = 0.38 *(n = 35)*

^a^ Numbers of relapses per year.
